# Expert Perspectives on Integrating Palliative Care into Primary Health Care: A Qualitative Analysis of a Modified Delphi Study

**DOI:** 10.3390/nursrep16010020

**Published:** 2026-01-09

**Authors:** Carolina Muñoz Olivar, Francisca Marquez-Doren, Juan Sebastián Gómez Quintero, Carla Taramasco Toro, Carlos Javier Avendaño-Vásquez

**Affiliations:** 1 Health Sciences, Faculty of Medicine, Antonio Nariño University, Bogotá 111321, Colombia; 2 Center for Cancer Prevention and Control (CECAN), Santiago 8331150, Chile; carla.taramasco@unab.cl; 3 Escuela de Enfermería, Facultad de Medicina, Pontificia Universidad Católica de Chile, Capítulo Alpha Beta Omicron de Sigma, Santiago 7820436, Chile; fmarquez@uc.cl; 4 Faculty of Engineering, Andrés Bello University, Santiago 8370211, Chile; juan.gomez@unab.cl; 5 Institute of Technologies for Innovation in Health and Well-Being, Faculty of Engineering, Andrés Bello University, Viña del Mar 8370211, Chile; 6 Escuela de Enfermería, Universidad Industrial de Santander, Bucaramanga 680001, Colombia; cjavevas@uis.edu.co

**Keywords:** palliative care, primary health care, health systems integration, qualitative research, expert consensus, nursing

## Abstract

**Background:** Integrating palliative care (PC) into primary health care (PHC) is essential for achieving Universal Health Coverage and reducing avoidable suffering. Despite global progress in PC development, the extent to which PC is effectively embedded within PHC systems remains unclear, particularly in low- and middle-income countries. Colombia illustrates this gap, with an advanced legal framework but persistent territorial inequities. This study explored how national experts conceptualize PC integration into PHC to inform the development of context-sensitive indicators. **Methods:** A directed thematic analysis was conducted using qualitative comments from a modified Delphi process (pre-Delphi, Round 1, Round 2). Coding was guided by the WHO model for PC development and the WHO–UNICEF Operational Framework for PHC, combining deductive and inductive approaches to identify recurrent themes. **Results:** A total of 230 qualitative comments from experts in PC, PHC, and public health were analyzed. Experts described integration as the alignment of policy, education, service delivery, and community participation within PHC structures. They emphasized that laws and training programs alone are insufficient; integration depends on implementation capacity, equitable access, and locally responsive systems. Rural areas were identified as facing the greatest barriers, including limited trained staff, restricted medicine availability, and weak referral pathways. **Conclusions:** Experts understood PC integration into PHC as a dynamic and ethical process linking system design with human experience. Strengthening equity, workforce preparation, and community engagement is essential to translate policy into practice and to develop meaningful indicators for health system improvement.

## 1. Introduction

Palliative care (PC) seeks to prevent and relieve the suffering associated with life threatening conditions. Although it is recognized as an essential part of health care, millions of people around the world still die each year with untreated pain or distress [1]. The World Health Organization (WHO) and UNICEF have emphasized that integrating PC into primary health care (PHC) is essential to achieving Universal Health Coverage and the Sustainable Development Goals [2,3]. Strong PHC systems reduce health inequalities by providing continuous, comprehensive, and community based care close to where people live.

In 2021, the WHO reached a global consensus on the indicators used to measure PC development and proposed an updated conceptual model that expanded the original domains. This framework identifies six interdependent areas for PC development: empowerment of people and communities, health policies, research, essential medicines, education and training, and integrated health service delivery [4]. These domains have guided important progress in PC worldwide [4,5,6]. However, development does not necessarily mean integration. The existence of national laws, programs, or strategies does not guarantee that PC is effectively embedded in PHC practice or that it reaches people beyond major cities or outside the formal insurance system. This gap between development and integration remains a major barrier to achieving equitable and person centered care [7].

Measuring the degree of integration is therefore crucial. Without valid and context sensitive indicators, governments cannot identify where policies succeed or fail, or where resources should be directed to reduce suffering and improve access. Integration indicators make it possible to connect structure and function, linking national policies and professional training with the actual experiences of patients and families. However, existing indicator frameworks such as those proposed by the WHO and the International Association for Hospice and Palliative Care (IAHPC) remain focused on system development rather than on how PC is implemented within PHC processes [5,7]. Consequently, the extent to which PC is effectively integrated into PHC systems remains poorly understood, particularly in low and middle income countries.

Colombia offers an illustrative example. The country has made considerable progress in PC policy and legislation, most notably through the Consuelo Devis Saavedra Law of 2014, which guarantees the right to access palliative care. More than twenty additional regulations, including laws, resolutions, and technical guidelines, govern its provision, financing, education, and the availability of essential medicines. However, this regulatory framework has not yet translated into full and equitable integration across the health system. Many patients in rural areas or with limited resources continue to face serious barriers, including lack of essential medicines, trained personnel, and referral mechanisms. According to the Global Palliative Care Development Score (GDS), Colombia is classified as having Established Palliative Care Development, with a score of 2.607, below El Salvador (2.750), Brazil (2.714), and Chile (3.321) [8]. This intermediate level reflects both the country’s progress in building a regulatory foundation and the persistence of territorial inequalities in access and service delivery [8]. Understanding and measuring how PC aligns with PHC in this context is therefore not only a scientific need but also an ethical responsibility.

This study examines the qualitative comments of a national panel of experts to understand how they conceptualize the integration of palliative care into primary health care in Colombia. This qualitative perspective provides contextual evidence that complements international metrics and supports the design of indicators that are both sensitive to local realities and useful for guiding equitable health system strengthening.

## 2. Materials and Methods

### 2.1. Study Design

This qualitative study used a directed thematic content analysis to explore how experts conceptualized the integration of PC within PHC systems. The qualitative material consisted of free-text comments gathered during a modified two-round Delphi with a pre-Delphi stage. The aim was to identify a set of indicators capable of assessing the degree of PC integration in PHC from a qualitative perspective, complementing the panel’s quantitative ratings.

### 2.2. Theoretical Framework

The analysis was guided by two global frameworks: (1) the WHO model for palliative care development [4], which includes six domains (policies, essential medicines, service delivery, education, research, and empowerment); and (2) the WHO–UNICEF Operational Framework for PHC [7], which organizes health systems into structures/inputs, processes, results, and impact. These models provided a single deductive scaffold for coding and interpretation, while remaining open to data-driven nuances that refined or contextualized the predefined categories. A flow diagram illustrating how both frameworks were integrated during the coding and analytical process is provided as Supplementary Materials Figure S1.

### 2.3. Expert Panel

Experts were recruited via email invitations disseminated through professional associations, universities, health institutions, national and regional health authorities, and PC networks. A snowball strategy complemented the initial outreach.

Eligibility: Participants were health professionals with ≥1 year of experience in PC, PHC, or public health, and meeting at least one of the following: membership in a national PC scientific society; current employment at a higher-education institution; position at the Ministry of Health or a regional health authority related to PC; involvement in public health policy; or service as national delegate to WHO/PAHO.

Language and consent: All Delphi materials and communications were in Spanish. Participants provided electronic written informed consent prior to enrolment. No compensation was offered.

### 2.4. Setting

The study was conducted in Colombia, with nationwide recruitment to ensure regional, and disciplinary diversity (PC, PHC, public health). Data collection occurred remotely through online surveys. The Delphi process included a pre-Delphi questionnaire followed by two iterative rounds (R1–R2) with structured feedback provided between rounds. All stages of data collection were conducted between March and December 2024. Experts were recruited from all seven national nodes that organize the 33 Colombian regions [9] (Caribbean, Pacific, Center, Orinoquia, Northeast, Amazonia, and Bogotá). This structure, established by the national palliative care network, ensured geographical representation and inclusion of different levels of health system organization. The diversity of nodes allowed the panel to incorporate perspectives from urban and rural contexts, public and private sectors, and both clinical and academic environments.

### 2.5. Data Collection

Each instrument included open comment fields allowing participants to justify, qualify, or refine their ratings for every candidate indicator of PC–PHC integration. Free-text comments were exported verbatim, anonymized, and organized by indicator, round, and participant code. Version logs documented data handling and typographical corrections. Quotes were analysed in Spanish; illustrative excerpts reported in English were translated by the research team and checked for semantic equivalence.

### 2.6. Data Analysis

We conducted a directed thematic content analysis [10], informed jointly by the WHO PC development model and the WHO–UNICEF PHC framework [4,7]. This dual framework operated as a single coding structure that integrates PC domains with PHC components (e.g., multisectoral policy/action, integrated services, results, and impact).

All free-text comments were anonymized and imported into Dedoose (version 9.0). Following Burnard’s systematic principles [11], analysis progressed through immersion, coding, reduction, and synthesis:

1. Data preparation: Compilation, anonymization, and organization of comments by indicator, round, and participant.
2. Framework-guided coding: Segmentation into meaning units and coding against the domain/component that best represented the content within the integrated WHO frameworks.
3. Refinement: When comments specified implementation aspects (e.g., audit mechanisms, funding execution, referral/continuity, rural access) these were retained as subcategories consistent with the frameworks. When a single expert comment addressed more than one conceptual domain or PHC component, the comment was segmented into discrete meaning units, and each segment was coded independently within the corresponding framework domain
4. Synthesis: Iterative comparison to develop subthemes that describe how experts contextualized and operationalized global recommendations within national PHC systems.
5. Consensus and audit: Two researchers (CMO, FMD) coded independently and resolved disagreements by discussion until consensus, maintaining an audit trail of codebook versions, analytic memos, and exemplar quotations.

Interpretive focus. The analysis emphasized integration points between PC domains and PHC results-chain components (processes, outputs/results, and impact).

Saturation and participant validation. Conceptual saturation was judged when no new framework-consistent dimensions emerged. Participant validation occurred implicitly through Round-2 feedback and re-rating.

The qualitative component of the modified Delphi was not intended to force full consensus, but rather to support structured and iterative expert reflection. Two Delphi rounds were considered sufficient, as no new thematic dimensions emerged during Round 2 and interpretative stability was observed across comments. This approach is consistent with the exploratory aim of the qualitative analysis, which focused on capturing how experts conceptualized integration, rather than on achieving numerical convergence.

### 2.7. Rigor and Reporting

Credibility was enhanced by triangulation across frameworks and analysts, dual independent coding, and peer validation of the category system. Dependability and confirmability were supported by a documented audit trail linking data segments to codes and interpretations. Transferability was supported by providing a detailed description of the study context and the expert panel, enabling readers to assess the relevance of the findings to other primary health care settings with similar characteristics. Reporting follows the 32-item COREQ checklist [12].

### 2.8. Ethical Considerations

This study complied with the principles of the Declaration of Helsinki. Ethical approval was granted by the Bioethics Committee of the Faculty of Medicine at Antonio Nariño University (Colombia), under Act Nº 13-2024, dated 21 August 2024. In addition, all qualitative data were anonymized prior to analysis and stored securely. Access to the dataset was restricted to the principal investigator. Digital files were stored on password-protected devices, with backups maintained on an offline computer not connected to external networks. No identifiable personal information was included in the analysis or reporting.

## 3. Results

A total of 30 experts participated in the pre-Delphi stage, 27 in Round 1, and 23 in Round 2, representing an 85% retention rate and a 15% attrition rate across Delphi rounds. The qualitative analysis included all written comments provided at each stage, resulting in 230 analyzable comments. The panel included a diverse group of professionals with experience in PC, PHC, and public health. Most participants were nurses (50%, *n* = 15), followed by physicians (37%, *n* = 11), and other health professionals such as social workers (*n* = 2), psychologists (*n* = 1), and spiritual counselors (*n* = 1) (13%). The average age was 40 years, ranging from 25 to 62. In terms of professional experience, 65% (*n* = 15) reported more than five years, 26% (*n* = 6) between two and five years, and 9% (*n* = 2) between one and two years. The experts represented the seven national nodes that group Colombia’s 33 regions, ensuring coverage of the Caribbean, Pacific, Central, Orinoquia, Northeastern, Amazon, and Bogotá areas. Most worked in academic, clinical, or community settings, often combining teaching, research, and service management. In addition, participants reported experience across three broad functional domains: clinical care (including primary and specialized palliative care delivery), academic roles (teaching and research), and administrative or policy-related functions (such as health management, quality improvement, project management, and community-based work), with several experts contributing perspectives across more than one domain. Participant identifiers were assigned at enrolment and maintained across Delphi stages; therefore, not all IDs appear in the qualitative excerpts, as some participants did not provide free-text comments in later rounds or their comments were not illustrative for reporting purposes. The analysis provided a comprehensive understanding of how experts conceptualized the integration of PC into PHC systems in Colombia. Their reflections revealed both structural and human dimensions of integration, showing how participants linked policy, education, service organization, and community participation. The findings are presented according to two guiding frameworks: the WHO model for PC development and the WHO–UNICEF Operational Framework for PHC. Together, they allowed the integration of the six domains of PC development (policy, essential medicines, service delivery, education, research, and empowerment) with the PHC components related to multisectoral action, integrated services, outcomes, and impact.

Table 1 presents illustrative quotations from experts, showing how comments were coded across the main domains and subdomains of both frameworks. These examples highlight the conceptual and contextual diversity of the qualitative data used to construct the thematic synthesis.

### 3.1. WHO Framework for Palliative Care Development

#### 3.1.1. Education and Training

Education emerged as a structural foundation of integration. Participants emphasized the importance of professional training that combines PC principles with PHC approaches. One comment noted, “Knowing whether health professionals have access to this type of training helps identify available human resources, but it would be more meaningful to measure outcomes: how many of those trained actually work in PC or PHC settings” (Id_02). Experts also pointed out informational gaps and regional inequalities in access to education, suggesting that “there is a general limitation in the quality of information on training coverage, which affects feasibility; this could be improved through a national repository of educational programs” (Id_12).

#### 3.1.2. Research

Research was viewed as a domain where integration between academic production and primary care practice remains limited. Experts agreed that the mere existence of research groups does not imply integration. As one participant observed, “The relationship is not clear to me; the existence of specialized professionals or associations does not necessarily demonstrate integration” (Id_02). Others stressed that research indicators should reflect how knowledge contributes to service improvement and patient care.

#### 3.1.3. Essential Medicines

Access to essential medicines, particularly opioids, was considered a tangible expression of integration. Several experts questioned the validity of current measurement approaches. One participant commented, “The indicator (pharmacies available 24/7) should go beyond opening hours and include real availability and responsiveness to prescribed medications” (Id_18). Another added, “It should be more specific to the drugs most used in PC and assess whether timeliness or quantity is more relevant” (Id_19). Together, these reflections positioned access to medicines as both a technical and ethical measure of system reliability.

#### 3.1.4. Health Policies

Participants recognized that national laws and strategies for PC are necessary but insufficient. They emphasized that integration requires implementation, monitoring, and adaptation to local contexts. One expert explained, “The existence of a national PC strategy is essential, but the indicator should reflect its implementation and real impact rather than its mere existence” (Id_02). Another noted, “An additional dimension that could strengthen the indicator is multisectoral and community participation in its formulation and updating, especially from PHC, to ensure that it reflects territorial needs and intercultural perspectives” (Id_18). These reflections highlight policy not as a static structure but as a dynamic mechanism that connects regulation, participation, and accountability.

#### 3.1.5. Service Delivery and Needs Assessment

This domain concentrated most of the discussion, linking service organization with access and equity. Experts debated how integration should be measured in diverse contexts. One participant suggested, “Define how access is measured. This is a super-indicator that could lead to others, considering availability, quality, and staff preparedness for PC within PHC” (Id_02). Another remarked, “Being insured does not guarantee access to PC; it is essential to know whether insurance actually includes these services” (Id_04). Others called attention to people outside the social security system, stressing that “it is important to include those who access services privately, not only those affiliated with a health regime” (Id_15).

Experts concluded that integration should be understood as the alignment between service design, social protection, and real accessibility in the first level of care. Several comments emphasized that this alignment remains particularly weak in rural and remote areas, where the availability of trained personnel, essential medicines, and referral mechanisms is limited. For these participants, achieving integration means ensuring that people living in rural territories have the same opportunities for relief of suffering as those in urban centers. Equity, therefore, was not viewed as a separate outcome but as the ultimate expression of integration.

#### 3.1.6. Empowerment of People and Communities

Community empowerment was seen as a central yet often neglected component of integration. Experts argued that patients and caregivers should be recognized as active participants rather than passive users of the health system. As one expert explained, “It is appropriate to identify indicators of PC integration within PHC processes, because even if this area is considered relevant, there is still much to improve, including the acceptance of PHC by patients and families and their understanding of the dying process” (Id_16). Another participant proposed strengthening feedback mechanisms: “A national survey including patients, families, caregivers, and health professionals would provide comprehensive feedback on the quality of palliative care and help adjust services to real needs” (Id_18).

### 3.2. Components of the Primary Health Care Framework

#### 3.2.1. Population Health Impact

Experts questioned whether mortality or prevalence indicators can truly reflect integration. One participant explained, “The prevalence and mortality of chronic diseases may indicate PC needs but not necessarily integration” (Id_04). Another added, “We should identify which programs have a PHC approach and measure their real impact through the graduates who work, lead, or legislate with that perspective” (Id_02). Mortality indicators were thus seen as indirect and context dependent measures.

#### 3.2.2. Impact on Suffering Reduction and Avoidable Mortality

Mortality was acknowledged as an incomplete measure of PC outcomes. Experts agreed that death rates do not capture relief of suffering, which is the central aim of PC. As one participant stated, “Although mortality is important, it does not show the true impact of palliative care, whose purpose is to relieve suffering rather than prevent death” (Id_03). Participants recommended complementing mortality data with indicators of quality of life and symptom control.

#### 3.2.3. Outcomes and Burden of Disease

Indicators related to disease burden were considered useful for estimating needs but insufficient to represent integration. One expert explained, “This indicator allows an approximation of PC needs but does not measure the integration between PC and PHC” (Id_05). Others recommended pairing these indicators with measures of continuity and coordination to better reflect how services respond to patient needs.

#### 3.2.4. Integrated Services and Equity

Equity emerged as a cross-cutting concern. Experts argued that integration cannot be claimed without equitable access across territories. One participant summarized, “Existing coverage data should be complemented with variables that measure real use and continuity of PC services, especially among vulnerable populations” (Id_18). Integration was described as both a technical and ethical challenge that requires building coherent and sensitive systems capable of reaching all communities.

### 3.3. Overall Synthesis

**Figure 1:**
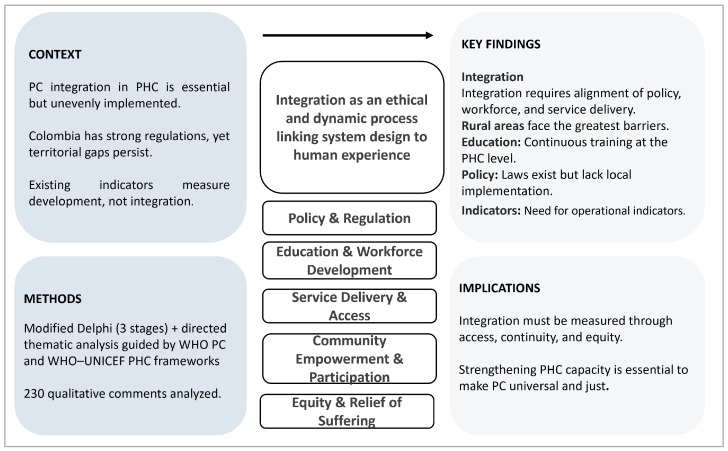
Conceptual model of palliative care integration within primary health care in Colombia. Legend: This conceptual model summarizes how experts described the integration of palliative care (PC) into primary health care (PHC) in Colombia. The model depicts integration as an ethical and dynamic process that links system design to human experience. It illustrates how policy, workforce development, service delivery, community participation, and equitable relief of suffering interact within PHC structures, reflecting the thematic synthesis derived from the qualitative analysis. Figure created by the authors.

Overall, experts described integration as a gradual process that connects policy, education, research, and service delivery under the ethical imperative of equity in care. Rather than a final outcome, integration was portrayed as a living process that aligns system structures with the relief of suffering and the participation of people and communities. These findings show how global frameworks can be contextualized within national health systems, transforming integration from a conceptual aspiration into a shared responsibility. In Figure 1, the conceptual model derived from the thematic analysis is presented. It visually summarizes how experts understood the integration of PC within PHC systems in Colombia. Integration is depicted as an ethical and dynamic process linking system design to human experience, progressing from policy and workforce development to service delivery, community participation, and the equitable relief of suffering.

## 4. Discussion

This qualitative analysis explored how experts conceptualized the integration of PC into PHC systems using two complementary frameworks developed by the WHO and UNICEF. Through their written reflections, experts described a gradual shift in the understanding of integration. It moved from focusing on the existence of laws, strategies, and service structures toward a concern with how these elements are implemented and monitored in local contexts. This change aligns with the argument of Vargas-Escobar et al. [13], who emphasize that integration should be observed in practice through mechanisms such as audit systems, fund execution, and service directories rather than only in policy texts.

Experts viewed integration as a balance between system design and the human experience of care. They associated it with ethical responsibility, continuity, and equity more than with isolated technical achievements. This interpretation is consistent with international literature describing integration as a multidimensional process that aligns the six pillars of PC development with the operational levels of PHC [4,7]. The emphasis on equity and community participation reflects the humanistic basis of both frameworks, where the relief of suffering cannot be separated from social justice.

The findings show that integration cannot be achieved merely through the existence of services, trained professionals, or essential medicines. It depends on how these components interact within PHC structures to ensure that relief of suffering is accessible to all. For the experts, the key question was not whether PC services exist but whether they reach those who need them, especially in rural or underserved areas.

From an international perspective, the challenges identified by Colombian experts are not unique. Countries with more consolidated experiences in integrating palliative care into primary health care, such as the United Kingdom and Australia, have shown that legal frameworks and service availability alone are insufficient to ensure effective integration [3,14,15]. In these settings, progress has depended on sustained investment in primary palliative care competencies, clear referral pathways between levels of care, and the incorporation of palliative care principles into routine primary health care practice rather than on isolated specialist services.

In Latin America, countries such as Chile and Brazil have also advanced in incorporating palliative care into primary health care through national strategies and training initiatives. However, regional and international assessments continue to document important territorial and organizational gaps, particularly in rural and underserved areas [9,16,17,18]. In this context, the contribution of the present study is not to compare countries or levels of development, but to offer a conceptual and indicator-based approach that makes integration visible within primary health care systems, supporting context-sensitive monitoring and decision-making [4,18].

### 4.1. Reinterpreting Global Frameworks in Local Contexts

Eighteen years after the launch of the Public Health Strategy for Palliative Care [19], the global development of PC has advanced significantly [4,5,6]. However, the indicators currently used to monitor this progress remain insufficient to measure integration with PHC. Experts agreed that these indicators describe the maturity of systems but do not capture how PC interacts with PHC processes. Integration requires going beyond counting services or measuring opioid consumption. It implies assessing how services operate as part of a coordinated system that provides equitable, continuous, and person-centered care.

This perspective supports the WHO–UNICEF position that effective public health strategies must be embedded across all system levels and appropriated by the communities themselves [7,20]. Experts’ comments reflected this principle: integration must be lived, not only declared. A health policy gains meaning when it improves access, continuity, and quality of care where people actually live.

### 4.2. Health Policy and System Transformation

Experts identified the gap between policy and practice as the main barrier to integration. Despite Colombia’s advanced legal framework for PC, its translation into operational mechanisms remains uneven across regions. Similar situations have been reported in Brazil, where reforms to the national primary care policy and budget reductions have weakened community-level implementation of PC [16]. In both countries, experts recognized that integrating PC into PHC requires more than professional training or family support. It demands a shift from curative biomedical models to a public health logic centered on social protection and equity.

### 4.3. From Development to Integration

The trajectory of PC development has laid the groundwork for integration but has not achieved it fully. According to experts, traditional indicators of development, such as the existence of laws or opioid consumption, represent the foundation rather than the goal. Integration requires measuring continuity, coordination, and effective coverage. The experts’ reflections complement the Brief Manual of Global Palliative Care Indicators [5], which recognizes that historical metrics capture development but not integration. By placing these measures within PHC, our findings fill this conceptual gap and highlight the need for indicators that identify bottlenecks, guide decisions, and direct investments toward equitable service expansion.

### 4.4. Equity and Rural Access

Equity emerged as a central theme across all domains. Many experts described the differences between urban and rural regions as the clearest sign of incomplete integration. The lack of trained personnel, opioid supply chains, and referral mechanisms in rural areas remains a structural weakness that limits the universality of care. This observation is consistent with evidence showing that strong PHC systems are the most effective way to reduce inequalities in access through local and continuous care [2,21,22]. As one expert noted, even economic capacity does not guarantee access when services are absent in remote territories. For integration to be meaningful, the place where people live should not determine the quality or availability of end-of-life care.

### 4.5. Education and Workforce Development

Education was understood as both a condition and a vehicle for integration. Experts highlighted the inclusion of PC content in undergraduate programs in nursing and medicine and the growth of postgraduate training. However, they also noted regional disparities and the need for continuous education for primary-level providers. This aligns with regional calls to strengthen competencies in primary palliative care (PPC), defined as PC provided by non-specialist professionals in community and PHC settings [14,17,18]. Strengthening these skills can improve quality of life and reduce the emotional and financial burden on families, especially in rural or underserved areas [3,15,23]. The experts’ reflections reinforce the idea that PC should not be treated as an isolated specialty but as a cross-cutting function within health systems.

Research was identified as the weakest link in the integration chain. Participants observed that many research initiatives remain disconnected from practice and that their impact is rarely measured. This gap has been recognized internationally as a persistent challenge for translating evidence into better care [24]. Strengthening research capacity within PHC settings could produce data that are more relevant for evaluating integration and shaping national strategies.

### 4.6. Integration as a Social Commitment

Beyond its technical dimension, integration was seen as an ethical and social responsibility. Experts linked it to the principles of the Alma-Ata and Astana Declarations, reaffirming health as a human right and community participation as essential for equity. This view connects PC integration with the Sustainable Development Goals and the Primary Health Care 30-30-30 Commitment for Universal Health Coverage [22]. In this sense, integration becomes both a moral imperative to relieve avoidable suffering and a governance task to ensure that no one is excluded from care.

### 4.7. Colombia’s Position in the Regional Context

Colombia is an upper middle income country with a decentralized administrative structure composed of 32 departments and one capital district. This organization contributes to marked territorial heterogeneity in health system capacity. Although more than 20 national regulations support the development of palliative care, persistent socioeconomic and geographic inequalities continue to shape uneven implementation across regions. These disparities, reflected in a high national Gini index, provide important context for understanding the emphasis placed by experts on equity, rural access, and implementation capacity within primary health care. Colombia’s progress in legislation and PC development provides a strong foundation for integration. The 2014 Consuelo Devis Saavedra Lawestablished PC as a national right, requiring education, service provision, and opioid availability. According to the Quality of Death Index (2015, 2021), Colombia moved from being unranked in 2010 to 42nd among 81 countries [24,25]. This improvement reflects early political action but also reveals persistent inequalities between regions and social groups. Compared with leading countries such as the United Kingdom, Australia, and Taiwan, Colombia’s challenge is not policy absence but implementation, funding, and rural coverage. Strengthening integration within PHC could be the next strategic step to translate policy into lived experience.

### 4.8. Policy and Practice Implications

For policymakers, these findings highlight that monitoring integration requires indicators that link structure, process, and outcome. Future tools should measure not only legal or structural aspects but also operational dimensions such as audit systems, budget execution, referral continuity, and community participation. For PHC teams, developing simple screening instruments to identify palliative needs early could facilitate timely referral and shared care across levels. Incorporating PC principles into education, service organization, and information systems can make integration both measurable and achievable.

### 4.9. Strengths and Limitations

A major strength of this study lies in its use of a directed thematic analysis based on two global frameworks, which ensured conceptual coherence and international comparability. The inclusion of experts from all seven national nodes provided regional diversity and strengthened the validity of interpretations. However, the analysis was limited by unequal participation across regions and by the lack of direct dialogue among experts, which is inherent to the Delphi design. The data were based on written reflections, which may have restricted more nuanced argumentation. Future studies could combine Delphi rounds with focus groups or interviews to deepen the interpretation. This study focused on experts’ perspectives and did not include patients or caregivers, whose voices should be incorporated in participatory designs.

## 5. Implications for Nursing Practice

Although this study did not focus specifically on nursing practice, the predominance of nursing professionals in the expert panel and the interpretation of their reflections allow consideration of implications for nursing within primary health care. The findings of this study suggest that nursing can play an important role in strengthening the integration of palliative care within primary health care, particularly from a public health perspective. Nurses often work closest to patients and families, which allows them to notice early signs of palliative needs and accompany people as they navigate the system. This proximity gives nurses the opportunity to support timely referral, clarify care preferences, and help maintain continuity when specialist services are limited.

Territorial differences described by the experts, especially those related to the availability of trained staff and essential medicines, reinforce the importance of preparing PHC nurses to respond to palliative needs in diverse settings. Increasing access to palliative care education, and ensuring that training is relevant to local realities, may help reduce gaps between urban and rural areas. Better preparation in communication, symptom assessment, and coordination of care has the potential to improve equity and strengthen the capacity of PHC teams.

The study also highlights the close connection between nursing practice and community engagement. Nurses often act as the link between health services and the social environment in which people live. Through health education, ongoing support for caregivers, and culturally sensitive communication, nurses contribute to public understanding of palliative care and help align it with the needs and values of each community. These activities support a PHC approach grounded in proximity, trust, and continuity.

In addition, the reflections of the experts suggest that nursing can contribute to monitoring how palliative care becomes part of everyday PHC practice. Because nurses participate in daily service organization, they are well positioned to observe practical barriers related to access, continuity, or referral pathways. Their involvement in local quality-improvement discussions can help ensure that the evaluation of integration reflects both system performance and the lived experience of patients and families. Through this role, nursing can support the development of indicators that are meaningful, feasible, and responsive to the reality of PHC settings.

## 6. Conclusions

Experts have understood the integration of palliative care into primary health care as a dynamic and ongoing process that connects policy, education, research, and service delivery under the principle of equity. Their reflections revealed that integration is not only a technical goal but also a moral and social responsibility. Aligning global frameworks with national contexts requires sustained commitment to community empowerment, equitable access, and the relief of suffering for all people.

## Figures and Tables

**Table 1:** Illustrative quotations from experts according to WHO and WHO–UNICEF frameworks.

Subdomain (WHO–UNICEF)	Illustrative Quotation	Delphi Stage	ID
**Palliative care development indicators**			
Education and training	“Knowing whether health professionals have access to this type of training helps identify available human resources, but it would be more meaningful to measure outcomes: how many of those trained actually work in PC or PHC settings.”	Round 1	02
	“There is a general limitation in the quality of information on training coverage, which affects feasibility; this could be improved through a national repository of educational programs.”	Round 1	12
	“It is important to identify who has training in PC but also in PHC, including teaching staff in medical and nursing programs.”	Round 1	16
	“A limitation in remote regions is the lack of virtual or continuous education opportunities in PC, which affects equity.”	Round 2	18
	“Continuous training in PC for physicians could strengthen integration with PHC.”	Round 2	30
Research	“The existence of specialized professionals or associations does not necessarily demonstrate integration; research indicators should reflect how knowledge contributes to service improvement.”	Round 1	02
	“It is important to examine not only the existence of research groups but also their production and impact in PC and PHC.”	Round 2	15
Essential medicines	“The indicator (pharmacies available 24/7) should go beyond opening hours and include real availability and responsiveness to prescribed medications.”	Round 2	18
	“It should be more specific to the drugs most used in PC and assess whether timeliness or quantity is more relevant.”	Round 2	19
	“The availability of opioids does not reflect the integrity or quality of access; details such as administration and continuity should also be evaluated.”	Round 2	19
	“The indicator of opioid consumption loses validity when considering that opioids are not the only medications used in PC.”	Round 2	30
Health policies	“The existence of a national PC strategy is essential, but the indicator should reflect its implementation and real impact rather than its mere existence.”	Round 1	02
	“An additional dimension that could strengthen the indicator is multisectoral and community participation in its formulation and updating, especially from PHC.”	Round 2	18
	“Having a law does not imply effective implementation; integration depends on how the regulation is applied at local levels.”	Round 1	05
	“It is important to identify whether the national law specifically supports PHC.”	Round 1	16
Service delivery and needs	“Being insured does not guarantee access to PC; it is essential to know whether insurance actually includes these services.”	Round 2	04
	“Define how access is measured. This is a super-indicator that could lead to others, considering availability, quality, and staff preparedness for PC within PHC.”	Round 2	02
	“Achieving integration is particularly challenging in rural areas, where trained staff, essential medicines, and referral systems are limited.”	Round 2	18
	“Hospital indicators are weak because they do not reflect the integration of PC with PHC.”	Round 2	30
	“It would be relevant to include differentiated indicators for pediatric and adult PC services.”	Pre-Delphi	21
	“Equity implies that people in rural territories should have the same opportunities for relief of suffering as those in urban centers.”	Round 2	18
Empowerment of people and communities	“It is appropriate to identify indicators of PC integration within PHC processes, because even if this area is considered relevant, there is still much to improve, including the acceptance of PHC by patients and families.”	Pre-Delphi	16
	“A national survey including patients, families, caregivers, and health professionals would provide comprehensive feedback on the quality of PC services.”	Round 2	18
	“Indicators for caregivers should be considered, as the caregiver–patient bond is indissoluble.”	Pre-Delphi	01
	“Community associations and patient groups are essential for advocacy and participation in PC.”	Pre-Delphi	21
**Primary health care framework**			
Population health impact	“The prevalence and mortality of chronic diseases may indicate PC needs but not necessarily integration.”	Round 1	04
	“We should identify which programs have a PHC approach and measure their real impact through the graduates who work, lead, or legislate with that perspective.”	Round 1	02
	“It would be relevant to study the burden of chronic diseases requiring PC, not only oncological ones, to estimate needs more accurately.”	Round 2	15
Suffering reduction/ avoidable mortality	“Although mortality is important, it does not show the true impact of palliative care, whose purpose is to relieve suffering rather than prevent death.”	Round 1	03
	“Mortality indicators are too general and should be complemented with measures of quality of life and symptom control.”	Round 2	19
Outcomes and burden of disease	“This indicator allows an approximation of PC needs but does not measure the integration between PC and PHC.”	Round 2	05
	“Burden of disease indicators should be combined with measures of continuity and coordination of care.”	Round 1	18
Integrated services and equity	“Existing coverage data should be complemented with variables that measure real use and continuity of PC services, especially among vulnerable populations.”	Round 2	18
	“Integration cannot be claimed without equitable access across territories.”	Round 2	19

Legend: The table presents representative quotations from Delphi experts illustrating how palliative care integration into primary health care was conceptualized according to the WHO Palliative Care Development framework and the WHO–UNICEF Operational Framework for Primary Health Care. Abbreviations: PC, palliative care; PHC, Primary Health Care.

## Data Availability

The original contributions presented in this study are included in the article. Further inquiries can be directed to the corresponding author.

## Supplementary Materials

The following supporting information can be downloaded at https://www.mdpi.com/article/10.3390/nursrep16010020/s1, Figure S1: Conceptual integration of the WHO Palliative Care Development Framework and the WHO–UNICEF Primary Health Care Operational Framework. The figure illustrates the deductive analytical scaffold used to guide coding and thematic synthesis, showing how palliative care development domains align with primary health care operational components, performance domains, and system objectives.

## Public Involvement Statement

No public involvement in any aspect of this research.

## Guidelines and Standards Statement

This manuscript was drafted in accordance with the Consolidated Criteria for Reporting Qualitative Research (COREQ) 32-item checklist for qualitative studies.

## Abbreviations

The following abbreviations are used in this manuscript:

GDS	Global Palliative Care Development Score
IAHPC	International Association for 50 Hospice and Palliative Care
PC	Palliative care
PHC	Primary health care
PPC	Primary Palliative Care
