# Supplementary figures and images for: Expert Perspectives on Integrating Palliative Care into Primary Health Care: A Qualitative Analysis of a Modified Delphi Study
Source: Nurs Rep. 2026 Jan 9;16(1):20. doi: 10.3390/nursrep16010020 (PMC12845302; doi:10.3390/nursrep16010020)

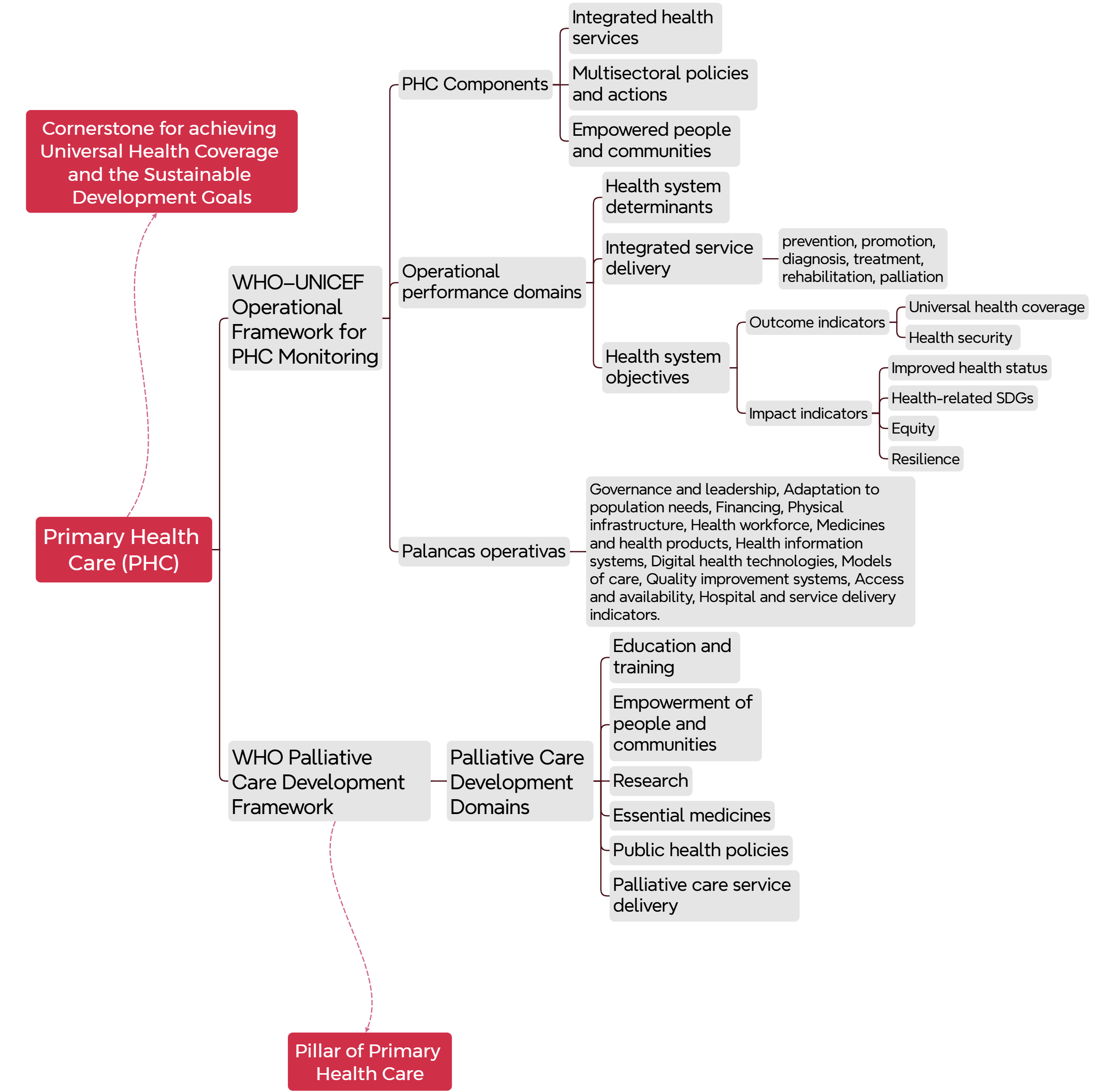

Supplement: Supplementary file 1 [file nursrep-16-00020-s001.zip › nursrep-4028116-supplementary.png]
